# Comprehensive analysis of the expression of N6-methyladenosine RNA methylation regulators in pulmonary artery hypertension

**DOI:** 10.3389/fgene.2022.974740

**Published:** 2022-09-12

**Authors:** Hao Zheng, Jing Hua, Hongpeng Li, Wenjuan He, Xiangyu Chen, Yingqun Ji, Qiang Li

**Affiliations:** Department of Pulmonary and Critical Care Medicine, Shanghai East Hospital, School of Medicine, Tongji University, Shanghai, China

**Keywords:** pulmonary hypertension, RNA methylation, m6A RNA modification, DEG analysis, ceRNAs

## Abstract

**Background:** Pulmonary arterial hypertension (PAH) is a progressive disease characterized by pulmonary vascular remodeling. The development of PAH involves N6-methyladenosine (m6A) modification. However, the functional role of m6A regulators in PAH and the underlying regulatory mechanisms remain unknown so far.

**Methods:** Microarray data (GSE149713) for monocrotaline induced PAH (MCT-PAH) rat models were downloaded and screened for differentially expressed genes (DEGs) and m6A regulators. Next, we screened for differentially expressed m6A regulators in endothelial cells (ECs), smooth muscle cells (SMCs), fibroblasts, interstitial macrophages, NK cells, B cells, T cells, regulatory T cells (Tregs) using scRNA sequencing data. The target DEGs of m6A regulators in ECs, SMCs, fibroblasts, and Tregs were functionally annotated using the Gene Ontology (GO) functional analysis and Kyoto Encyclopedia of Genes and Genomes (KEGG) pathway analysis. In addition, the cellular interaction analysis was performed to reveal the receptor—ligand pairs regulated by m6A regulators. Pseudo-time trajectory analyses were performed and a ceRNA network of lncRNAs-miRNAs-mRNAs was constructed in SMCs. Furthermore, the RNA transcriptome sequencing data for the SMCs isolated from idiopathic PAH (IPAH) patients (GSE144274) were validated for differentially expressed m6A regulators. Moreover, the HNRNPA2B1 levels in the lung samples from PAH patients and MCT-PAH were determined using immunohistochemistry.

**Results:** The m6A regulators were observed to be dysregulated in PAH. HNRNPA2B1expression level was increased in the PASMCs of scRNAs and IPAH patients. The target DEGs of HNRNPA2B1 were enriched in the regulation of muscle cell differentiation and vasculature development in PASMCs. The HNRNPA2B1 expression levels determined were consistent with the proliferation-related and collagen synthesis-related gene COL4A1. Moreover, the predicted transcription factors (TFs) foxd2/3 and NFκB could be involved in the regulation of HNRNPA2B1. HNRNPA2B1 might be regulating SMCs proliferation and phenotypic transition *via* rno-miR-330–3p/TGFβR3 and rno-miR-125a-3p/slc39a1. In addition, HNRNPA2B1 was observed to be highly expressed in the lung samples from MCT-PAH rat models and patients with PAH.

**Conclusion:** In summary, the present study identified certain key functional m6A regulators that are involved in pulmonary vascular remodeling. The investigation of m6A patterns might be promising and provide biomarkers for diagnosis and treatment of PAH in the future.

## Introduction

Pulmonary arterial hypertension (PAH) is a progressive disease characterized by persistent vasoconstriction and excessive vascular remodeling, leading to right heart dysfunction and a series of clinical symptoms. PAH has a poor prognosis, and no effective treatment methods are currently available for this condition which might prove to be fatal for patients with PAH (Humbert et al., 2018; Ruopp and Cockrill, 2022). Pathologically, the main pathophysiological features of PAH include the obstructive remodeling of small pulmonary arteries, peripheral vascular inflammation, and metabolic changes. Excessive proliferation, migration, and apoptosis resistance of the pulmonary artery endothelial cells (PAECs) and pulmonary artery smooth muscle cells (PASMCs) are the prominent features of vascular remodeling, and ECs dysfunction is an important initiating factor of vascular injury (Poch and Mandel, 2021). Recent studies have demonstrated the involvement of inflammation and immune disorders in pulmonary vascular remodeling, particularly through the secretion of cytokines, chemokines, metabolic reprogramming, etc., *via* the participation of macrophages and regulatory T cells (Tregs) (Humbert et al., 2004).

N6-methyladenosine (m6A) is a ubiquitous and abundant transcriptional modification. Existing studies report that m6A modification affects mRNA transport, degradation, translation, and metabolism, thereby being involved in a variety of pathophysiological processes (Qin et al., 2020). The m6A modification is a reversible modification that is regulated by the following three components—m6A methyltransferases serving as the “writers,” m6A demethylases serving as the “erasers,” and m6A binding proteins serving as the “readers” of the process (Tang et al., 2021b). Dysregulation of m6A reportedly leads to the occurrence and development of tumors, inflammatory diseases, immune system diseases, cardiovascular diseases, and metabolic disorders (Jiang et al., 2021b; Deng et al., 2022). Accumulating evidence indicates that the continuous dynamic modulation of the novel mediator m6A influences the expression levels of specific genes involved in several physiological and pathological processes occurring in PAH (Hu et al., 2021; Qin et al., 2021a; Xu et al., 2021).

For instance, Feng Chen et al. reported dysregulation of m6A and increased levels of YTHDF1 protein in pulmonary hypertension samples and hypoxic PASMCs. YTHDF1 reportedly promotes the phenotype switch in PASMCs proliferation and PAH development by enhancing the translation of MAGED1 in an m6A-dependent manner (Hu et al., 2021). Qi-Cai Wu et al. reported that the expressions of SETD2 and METTL14 were elevated in the PASMCs from hypoxia-induced PAH mice, while the lack of SETD2 in PASMCs attenuated the METTL14 expression levels and m6A RNA methylation levels, protected the mice from hypoxia induced pulmonary hypertension (HPH), and significantly reduced the right ventricular systolic pressure (RVSP), right ventricular/left ventricular plus septum [RV/(LV + S)] weight ratio, and pulmonary median width (Zhou et al., 2021b). In addition, METTL3 was abnormally upregulated, and YTHDF2 levels were significantly increased in the PASMCs. Furthermore, YTHDF2 recognized the METTL3-mediated m6A-modified PTEN mRNA and decreased PTEN levels, which led to the proliferation of PASMCs through the activation of the PI3K/Akt signaling pathway (Qin et al., 2021a). Yunbi Xiao observed that FTO and ALKBH5 were downregulated, METTL3 and YTHDF1 levels were increased, and m6A methylation was enhanced in the lung tissues of MCT-PAH rats. The coding genes associated with the up-methylation were primarily enriched in the inflammation, glycolysis, ECM–receptor interaction, and PDGF signal pathways, while the genes associated with down-methylation were related to the members of TGF-β family receptors (Zeng et al., 2021). The transcriptome-wide map of m6A circRNAs in HPAH was identified, which revealed that M6A could influence the circRNA-miRNA-mRNA network (Su et al., 2020). However, the roles of m6A regulators in PAH have not been elucidated so far. In order to develop effective interventions for PAH, it is particularly important to explore the mechanisms underlying the role of m6A regulators in PAH and determine the associated cell-specific regulatory network.

In this context, the present study aimed to explore the m6A regulators involved in PAH. The results revealed that m6A regulators exhibited differential expression in the lung samples of MCT-PAH rat models compared to the controls. Next, the differently expressed genes (DEGs) and m6A regulators in special cell types were analyzed using the single-cell RNA (scRNA) sequencing data. In addition, the functions of the DEGs of m6A regulators (the DEGs that overlapped with the target genes of m6A regulators) in PASMCs, PAECs, fibroblasts, and Tregs were identified. Furthermore, the role of m6A regulators in cellular communication was investigated, pseudo-time trajectory analysis in SMCs were performed and a ceRNA (HNRNPA2B1-lncRNA-miRNA-mRNA) regulatory network was constructed. Next, the regulation of HNRNPA2B1 through the transcription factors (TFs) in PASMCs was predicted. Furthermore, the expression of HNRNPA2B1 in the lung samples from MCT-PAH rat models and PAH patients was evaluated using immunohistochemistry. The present study involved a comprehensive analysis of m6A regulators in MCT-PAH lung samples, which provided important clues to identifying novel biomarkers to be used in the diagnosis and treatment of PAH.

## Materials and methods

### Data collection

The RNA sequencing data of monocrotaline-induced pulmonary arterial hypertension samples (*n* = 3) and controls (*n* = 5) were obtained from the GEO database (gse149713). The scRNA expression data (exprMatrix.tsv) and cell clustering information (meta.tsv) of the samples, which included the gene expression profile of six controls and 6 MCT–PAH lung samples, were downloaded from http://mergeomics.research.idre.ucla.edu/PVDSingleCell/. The human RNA sequencing data of four healthy and four idiopathic PAH (IPAH)PASMCs, each with a complete expression matrix, were obtained from the GEO database (gse144274). Related scripts could downloaded from https://github.com/TJZHENGHAO/scRNA-seq-and-m6A.

### Difference analysis for the identification of DEGs

The limma software v3.42.2 (Ritchie et al., 2013) was employed for conducting the differential analysis. The downstream analysis was conducted using *p*-value < 0.05 and |log2 (fold change) | > 0.322 as the criteria for gene screening. The correlation graphics data were presented in ggplot2 v3.3.4 (Randle and Zhuo, 2019).

### Association between the identified DEGs and the targets of m6A

In order to determine the functions of the m6A regulators, the potential target genes of these m6A regulators (rat) were downloaded from m6A2 Target (http://m6A2target.canceromics.org/#/download) and overlapped with the targets of m6A for the downstream analysis.

### Function enrichment analysis

ClusterProfiler v3.14.3 (Yu et al., 2012) bioinformatics resources were employed to conduct the Gene Ontology (GO) and Kyoto Encyclopedia of Genes and Genomes (KEGG) pathway annotations. The GO analysis revealed the enriched Biological Processes (BP), Cellular Components (CC), and Molecular Functions (MF). In addition, bubble charts were generated using the ggplot2 package of R software. The *p*-values were calculated based on the cumulative hypergeometric distribution. *p* < 0.05 was considered the threshold of significance.

### Protein–protein interaction analysis

A Protein–protein interaction (PPI) network was established using the online search tool STRING, with the combined score of ≥0.4 used as the criterion. The established PPI network was visualized in Cytoscape v3.7.2.

### Cell communication and pseudo-time trajectory analysis

Cellphonedb v3.0.0 (Efremova et al., 2020) was employed to conduct the cell communication analysis. Monocle v2.14.0 (Trapnell et al., 2014) was employed to conduct the pseudo-temporal analysis.

### Construction of a ceRNA (HNRNPA2B1-lncRNA-miRNA-mRNA) regulatory network

The interaction between HNRNPA2B1 and lncRNA was determined using RNAct (Lang et al., 2018). The interaction score represented the strength of the interaction, and the TOP 10 of the results were selected for the subsequent downstream analysis. The miRanda (Enright et al., 2003) software (v3.3a) was employed to match the lncRNAs and miRNAs. The target genes of the miRNAs were predicted using the RNAInter (Lin et al., 2020) (v3.0) database. Subsequently, the interactions between the miRNAs and lncRNAs or mRNAs were integrated to construct a ceRNA regulatory network, which was visualized in Cytoscape (Otasek et al., 2019).

### Predictive analysis of the interaction between target genes and transcription factors

The transcription factors that interacted with the target genes were predicted using JASPAR 2020 (Fornes et al., 2019) and TFBSTools (Tan and Lenhard, 2016) in the R package (non-default parameter: relScore = “85%”).

### Immunohistochemistry

Twelve rats were divided into the control and pulmonary hypertension group, six rats in each group. The animal model of pulmonary hypertension was established by injecting 6-week-old male Sprague-Dawley (SD) rats with Monocrotaline (MCT; Sigma-Aldrich) (60 mg/kg) intraperitoneally. In addition, control rats received normal saline (0.9% NaCl). After 3 weeks, the lung tissues from the rats were fixed with paraformaldehyde and subjected to the evaluation of HNRNPA2B1 expression (1:150, Abcam lot: 31,645) using immunohistochemical staining. The expression of HNRNPA2B1 in the lung tissue samples from three PAH patients and three healthy donors was also determined. Images were examined using Image-Pro Plus 6.0 software (Media Cybernetics, United States).

## Results

### The expression of N6-methyladenosine regulators was dysregulated in the MCT-PAH lung samples

**FIGURE 1:**
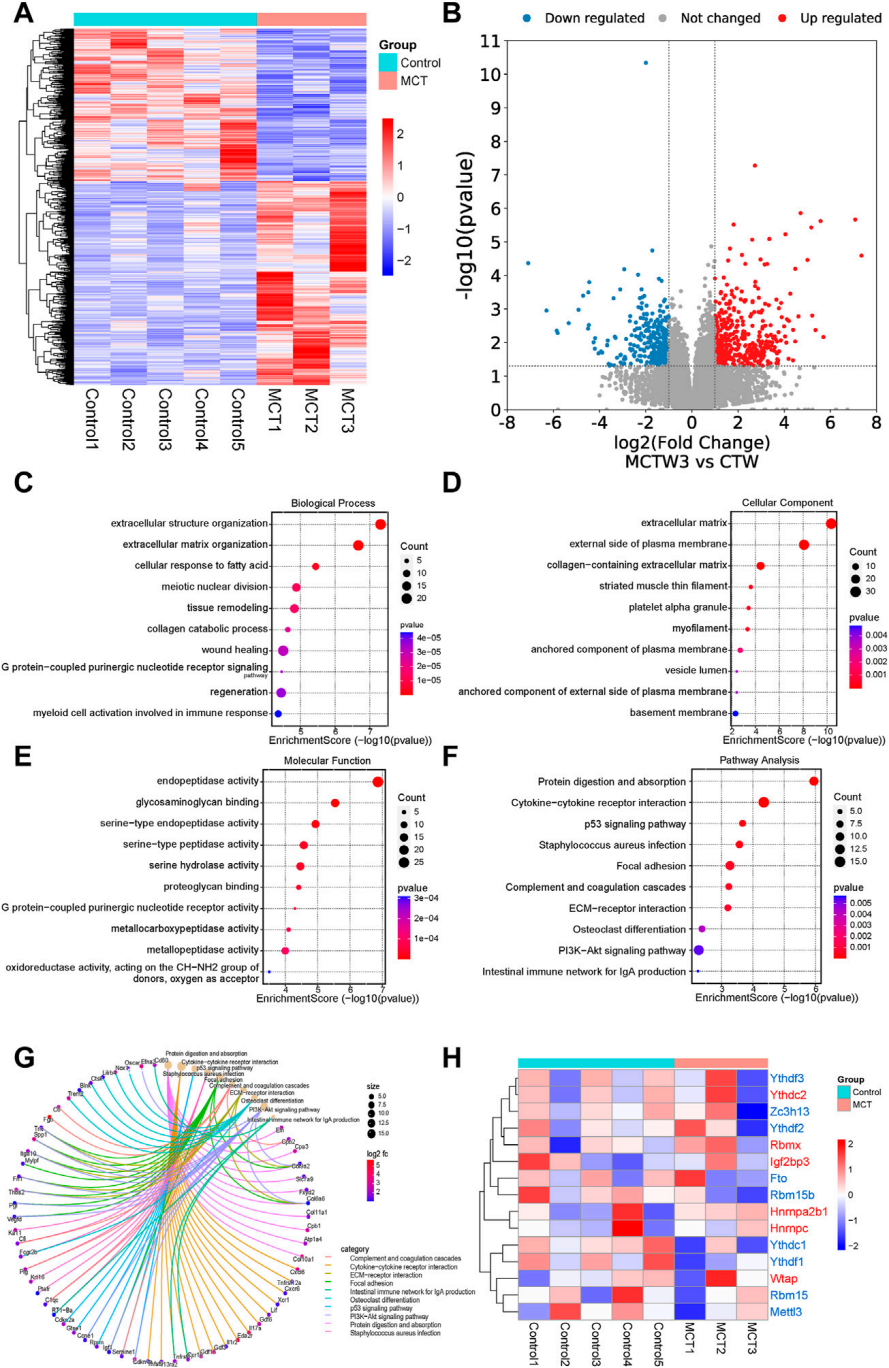
The RNA sequencing analysis revealed that the monocrotaline-induced PAH samples exhibited dysregulated expression of m6A regulators. **(A)** The heatmap and **(B)** volcano map revealed the DEGs in the MCT–PAH rat lung samples compared to the controls. The results of the GO analysis, **(C)** Biological process **(D)** cellular component **(E)** molecular function analysis revealed the potential signaling pathways regulated by the upregulated DEGs in the MCT groups compared to the controls. **(F)** Pathway analysis of the upregulated DEGs *via* KEGG analysis.**(G)** The genes that regulated the above functions are depicted. **(H)** The heatmap for the expressions of 15 m6A regulators in the two groups.

The expression profiles of the MCT–PAH rat models and controls were investigated (gse149713). The heatmap and volcano map presented in Figures 1A,B, respectively, illustrate the DEGs in the MCT–PAH rat lung samples compared to the controls. The identified DEGs were then subjected to GO and KEGG analyses, and the results are presented in Figures 1C–F, respectively. The overexpressed genes were enriched significantly in the cytokine receptor interaction, ECM−receptor interaction, focal adhesion, p53 signaling pathway, and PI3K−Akt signaling pathway, etc. The genes that regulated these functions are presented in (Figure 1G). Next, the expression of 15 m6A regulators was investigated, including methyltransferases (“writers”; METTL3, WTAP, RBM15), dedicated demethylases (“erasers”; FTO), and m6A RNA binding protein (RBP), YT521-B homology (YTH) domain, HNRNPA2B1 and insulin-like growth factor 2 mRNA-binding protein (IGF2BP) domain. The results (Figure 1H) revealed that HNRNPA2B1, HNRNPC, YTHDC2, WTAP, RBMX, and IGF2BP3 were increasing expressed, and YTHDC1, FTO, RBM15, RBM15B, METTL3, YTHDF1, YTHDF2, YTHDF3, and ZC3H13 expression were decreased in the PAH lung tissues compared to the controls, while, without significant difference. Therefore, analysis of specific -cell types is of great significance.

### The expressions of N6-methyladenosine regulators were dysregulated in PAECs

**FIGURE 2:**
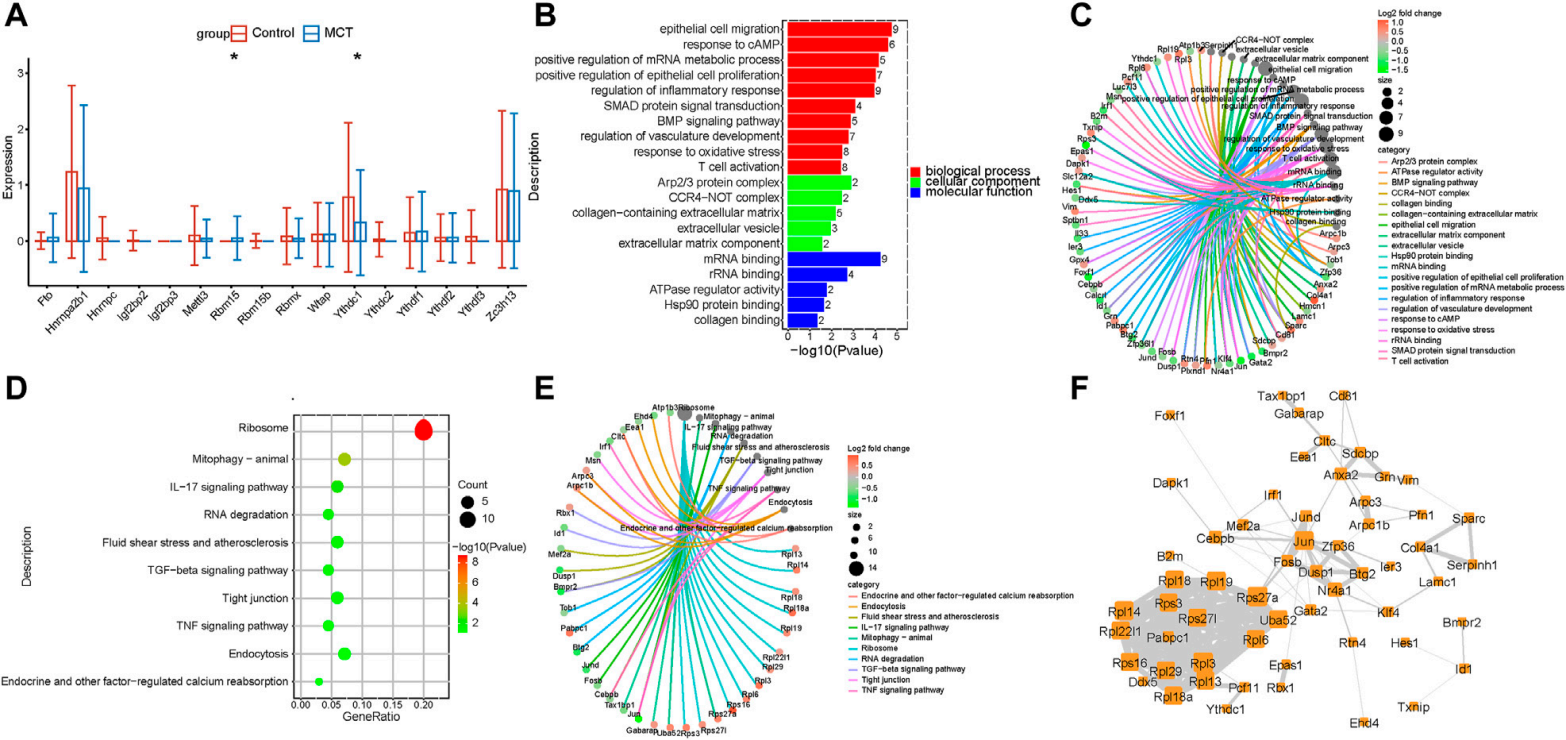
The expression of m6A regulators was dysregulated in PAECs. **(A)** The m6A regulators were expressed differentially in PAECs. **(B)** GO analysis of the target genes of YTHDC1 that overlapped with the upregulated DEGs. **(C)** The genes involved in the enriched pathways are depicted. **(D)** The KEGG analysis of the target genes of YTHDC1 overlapped with the upregulated DEGs. **(E)**The genes involved in the enriched pathways are depicted. **(F)** The hub networks constructed based on the genes presented above.

In order to investigate the m6A regulators and their functions in specific cell types of PAH, scRNA data analyses were performed. It was revealed that the m6A regulators were dysregulated in the PAECs. YTHDC1 was decreased while RBM15 was significantly upregulated in the MCT–PAH groups (Figure 2A). Next, to investigate the functions of the target DEGs of m6A regulators, the target genes of YTHDC1 were overlapped with the DEGs. Subsequently, the overlapped DEGs were subjected to KEGG and GO analyses. The results revealed that the overlapped DEGs were related to endothelial migration, response to cAMP, TNF signaling pathway, fluid shear stress and endothelial oxidative stress, etc. (Figure 2B and Figure 2D). The genes involved in different functions are presented in Figure 2C and Figure 2E. Furthermore, the above genes were used for constructing hub networks (Figure 2F).

### The expressions of N6-methyladenosine regulators were dysregulated in the PASMCs

**FIGURE 3:**
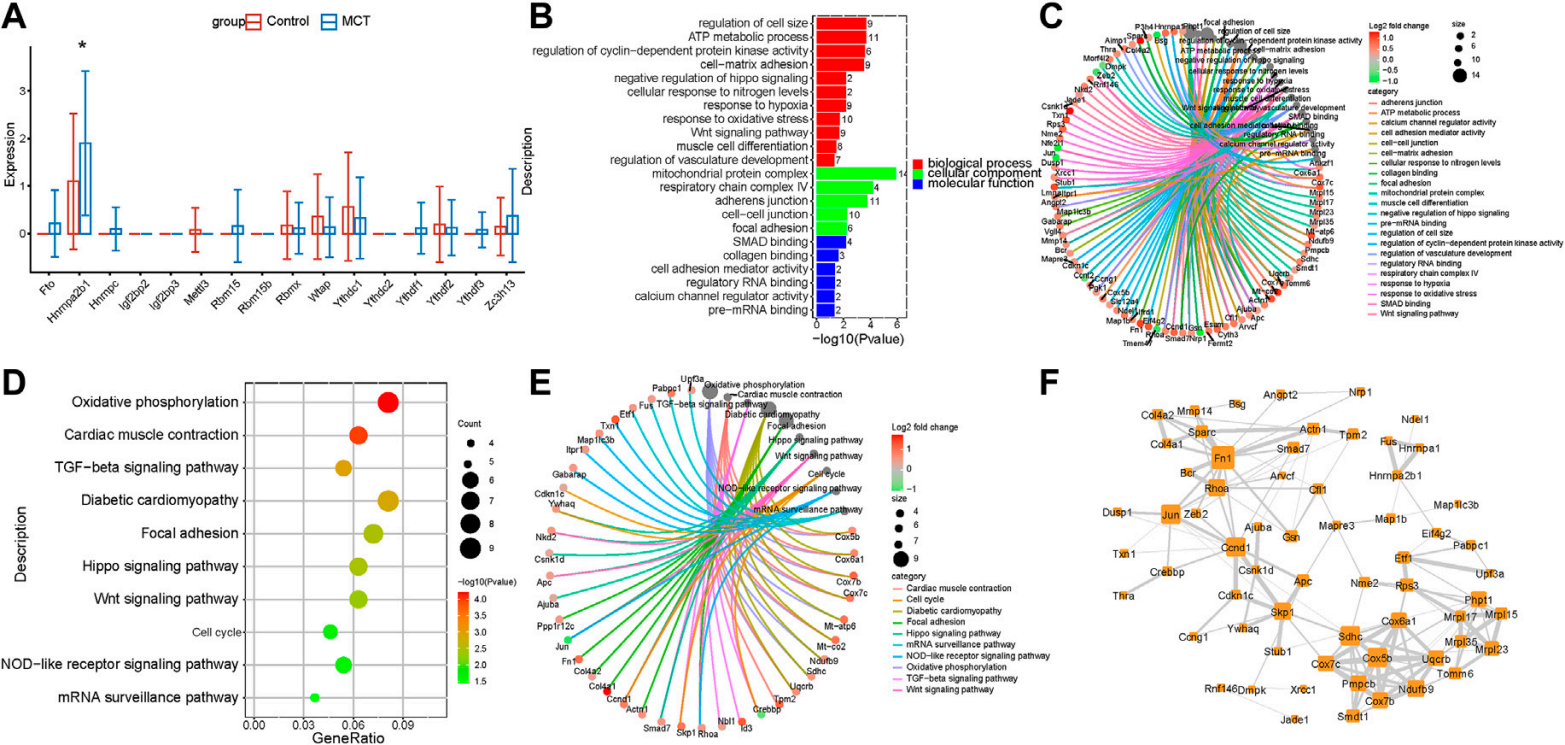
HNRNPA2B1 was overexpressed in PASMCs. **(A)** The m6A regulators were expressed differentially in the PASMCs. **(B)** The GO analysis of the target genes of HNRNPA2B1 that overlapped with the upregulated DEGs. **(C)** The genes involved in the enriched pathways are depicted. **(D)** The KEGG analysis of the target genes of HNRNPA2B1 overlapped with the upregulated DEGs. **(E)** The genes involved in the enriched pathways are depicted. **(F)** The hub networks constructed based on the genes depicted above.

Pulmonary artery vascular remodeling is a consequence of the conversion of quiescent contraction to proliferating synthetic SMCs. Studies have reported the involvement of m6A modification in the regulation of SMCs proliferation. The analysis conducted in the present study revealed a novel dysregulation of m6A regulators in PASMCs. HNRNPA2B1 was increased in the PASMCs, which was consistent with the results of lung transcriptome analysis (Figure 1H, Figure 3A). In order to investigate the function of HNRNPA2B1, the target genes of HNRNPA2B1 were overlapped with the upregulated DEGs, and the resulting overlapped DEGs were subjected to KEGG and GO analyses. The results revealed that HNRNPA2B1 participated in the Wnt signaling pathway, SMAD binding, cell cycle muscle cell differentiation, and TGF *β* signaling pathway *via* m6A modification (Figures 3B,D). The genes involved in the above functions are presented in Figures 3C,E. The above genes were also used for constructing hub networks and Ccnd1, Fn1, Skp1, Rhoa, and Smad7 were involved in the regulation of important functions (Figure 3F).

### The expression of m6A regulators was dysregulated in fibroblasts

**FIGURE 4:**
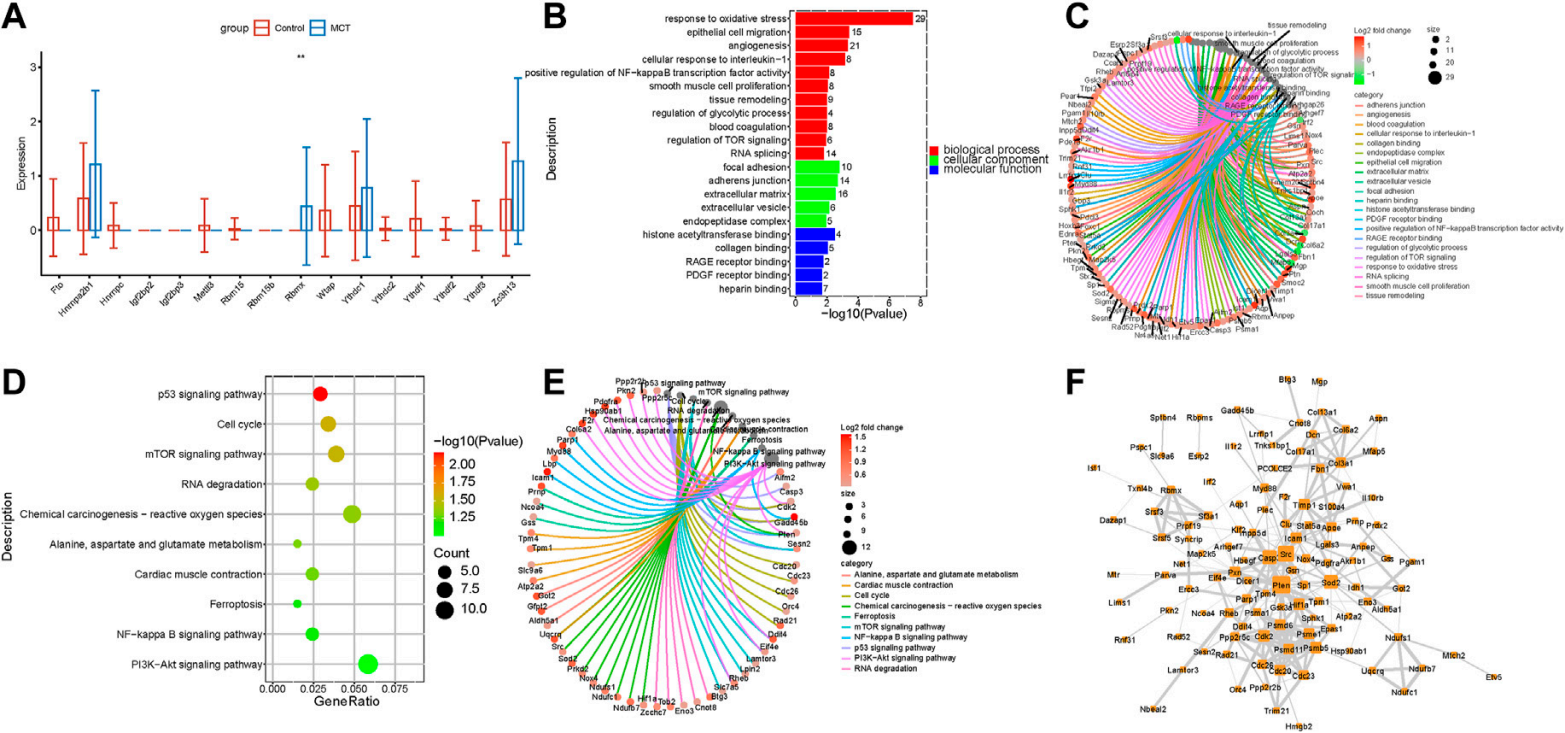
The expression of N6-Methyladenosine regulators was dysregulated in fibroblasts. **(A)** RBMX expression was increased in the fibroblasts. **(B)** The GO analysis of the target genes of RBMX that overlapped with the upregulated DEGs. **(C)** The genes involved in the enriched pathways are depicted. **(D)** The KEGG analysis of the target genes of RBMX overlapped with the upregulated DEGs. **(E)** The genes involved in the enriched pathways are depicted. **(F)** The hub networks constructed based on the genes depicted above.

Fibroblasts are activated through various pathways. Activated fibroblasts proliferate excessively during the pulmonary vascular remodeling in PAH. While the m6A regulators are reportedly involved in the regulation of tumor myofibroblast proliferation, their roles in pulmonary hypertension remain to be elucidated so far. The present study revealed that m6A regulators were dysregulated in the fibroblasts of the MCT—PAH groups compared to the controls. The RBMX levels were significantly increased (Figure 4A). The functional enrichment analysis of the intersecting genes between the DEGs and target genes of RBMX revealed that RBMX participated in the regulation of the glycolytic process, PDGF receptor binding, NFκB transcription factor activity, P53 signaling pathway and PI3K−Akt signaling pathways (Figures 4B,D). Previous studies have reported that these pathways are important for fibroblast activation and proliferation. Therefore, regulating the m6A regulators and targeting the m6A-modified genes could be a novel research direction in the field of translational studies. The genes involved in the enriched functions are presented in Figures 4C,E. In addition, the above genes were used for constructing hub networks and HIF1, PTEN, ICAM1, CDK2, and Col3a1were involved in the regulation of fibroblasts biological functions (Figure 4F).

### The expression of N6-methyladenosine regulators was dysregulated in the immune systems

**FIGURE 5:**
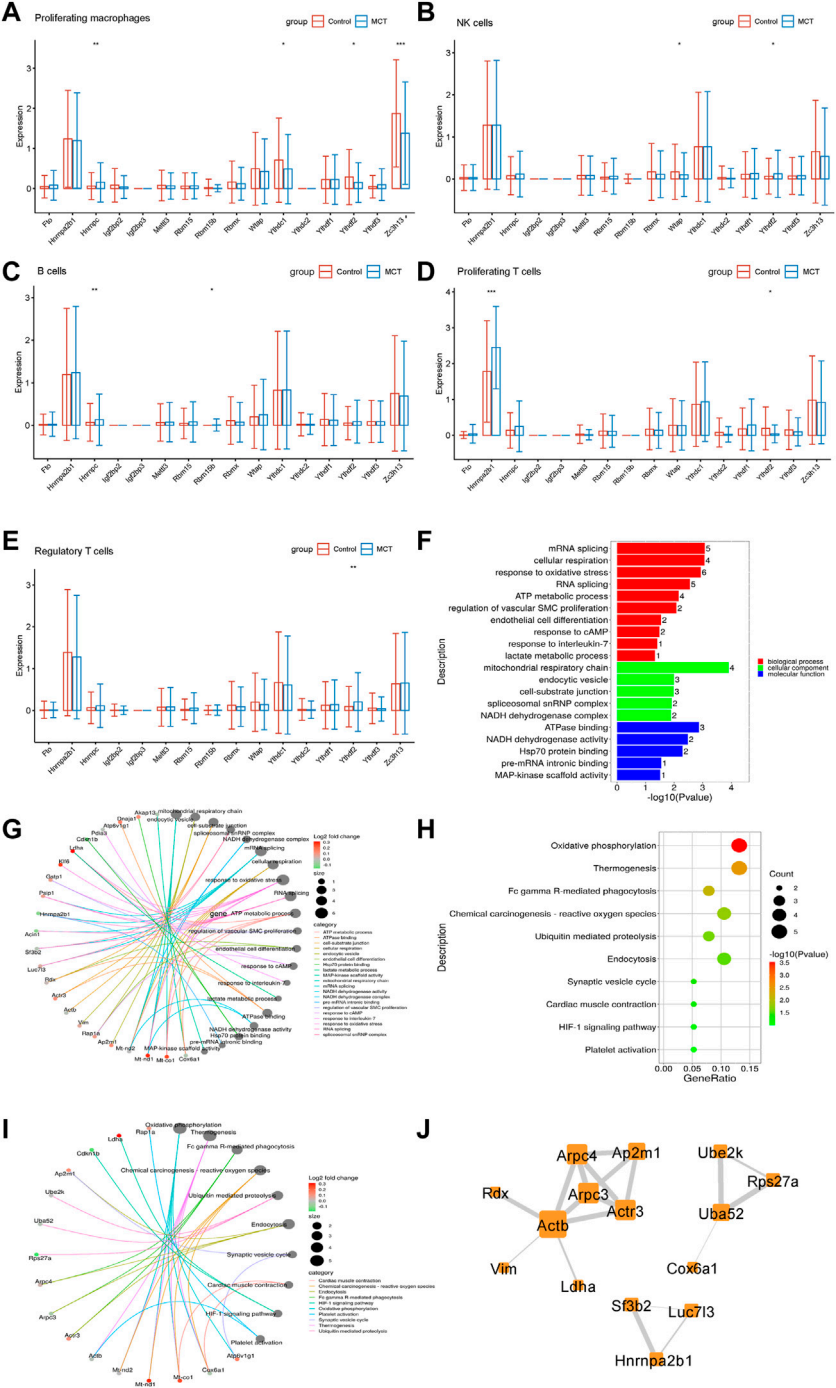
The expression of N6-Methyladenosine regulators was dysregulated in the immune systems. **(A)** The m6A regulators expressed differentially in interstitial macrophages. **(B)** The m6A regulators expressed differentially in NK cells. **(C)** The m6A regulators expressed differentially in B cells. **(D)** The m6A regulators expressed differentially in proliferating T cells. **(E)** The m6A regulators expressed differentially in Tregs. **(F)** The GO analysis of the target genes of m6A that overlapped with the upregulated DEGs in Tregs. **(G)** The genes involved in the enriched pathways are depicted in Tregs. **(H)** The KEGG analysis of the target genes of YTHDC1 overlapped with the upregulated DEGs in Tregs. **(I)** The genes involved in the enriched pathways are depicted in Tregs. **(J)** The hub networks constructed based on the genes depicted above.

The immune system may regulate pulmonary hypertension *via* multiple mechanisms (Tomaszewski et al., 2021). However, no studies have so far reported alterations in the levels of m6A regulators in the immune system in pulmonary hypertension. In the present study, m6A regulators were observed to be dysregulated in interstitial macrophages, NK cells, B cells, T cells, and Tregs. While HNRNPC levels were increased in interstitial macrophages, the levels of YTHDF2, ZC3H13, and YTHDC1 were decreased (Figure 5A). YTHDF2 levels were increased, and WTAP levels were decreased in NK cells (Figure 5B). HNRNPC and RBM15B levels were increased in B cells (Figure 5C). HNRNPA2B1 was increased in T cells (Figure 5D). YTHDF2 levels were increased in Tregs, while the levels of RBM15 were upregulated significantly in Tregs (Figure 5E).

Recent research has revealed that Tregs play an important role in the occurrence and development of PAH (Tomaszewski et al., 2021). Therefore, the role of m6A regulators in Tregs of MCT-PAH was explored in the present study. In order to investigate the function of m6A-modified DEGs, the target genes of m6A regulators were overlapped with the upregulated DEGs, and the resulting overlapped DEGs were subjected to KEGG and GO analyses. The results of these analyses revealed that the overlapped DEGs were related to the regulation of vascular SMCs proliferation, lactate metabolic process, response to oxidative stress, HIF-1 signaling pathway, and platelet activation, etc. (Figures 5F,H). The genes involved in these functions are presented in Figures 5G,I. These genes were then used for constructing hub networks and Arpc3, Actb, and Uba52 were involved in the regulation of important functions (Figure 5J).

### HNRNPA2B1 was involved in the regulation of vascular remodeling *via* cellular interaction and PASMCs phenotypic switch

**FIGURE 6:**
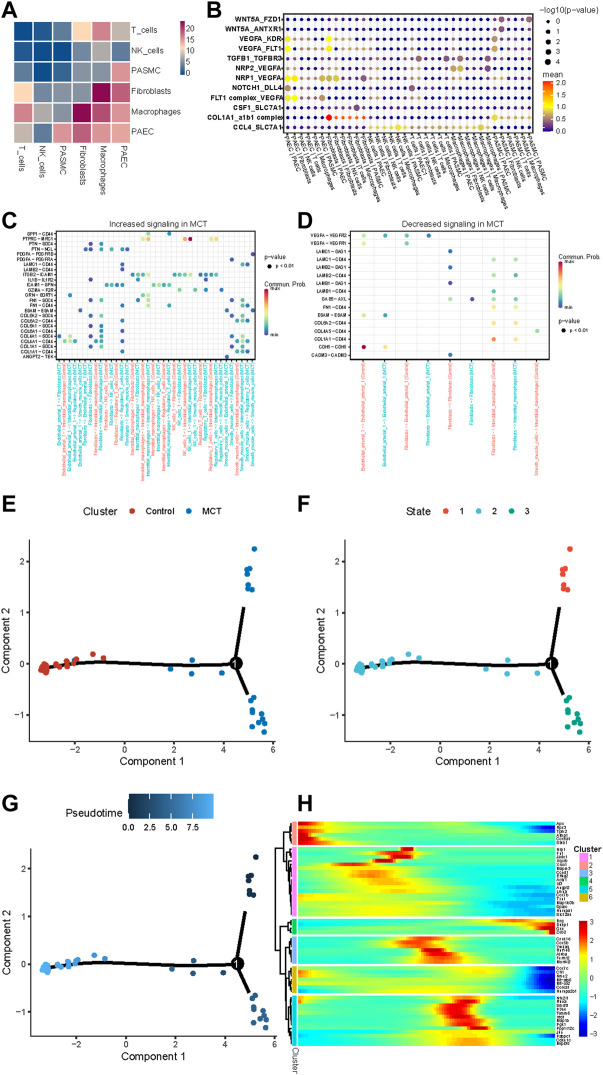
Results for the assessment of cell–cell Interactions in MCT-PAH and the pseudo-time trajectory analysis conducted with smooth muscle cells. **(A)** The cell–cell interactions in MCT-PAH involved 6 cell types. **(B)** The ligand–receptor pairs are presented. The cell type labels are designated as (the cell type expressing the ligand)—(the cell type expressing the receptor). The ligand-receptor pairs were regulated by HNRNPA2B1. **(C,D)** Increasing signaling and decreasing signaling in MCT are presented. **(E–G)** Pseudo-time trajectory analysis were performed in SMCs (cluster, state and Pseudo-time).**(H)**The gene expression revealed in the pseudo-chronological analysis conducted with SMCs.

Cellular communication analysis was performed on the scRNA dataset, which revealed interactions of SMCs with ECs, T cells, and fibroblasts (Figure 6A). Next, the receptor ligands were intersected with the target genes of HNRNPA2B1, and the results are presented in Figure 6B. Moreover, COL1A1/CD44, COL4A1/CD44, FN1/CD44, LAMC1/CD44, and COL6A2/CD44 signaling related to the interactions between interstitial macrophages and SMCs were increased in MCT-PAH compared with controls. Meanwhile, COL6A2/CD44, FN1/CD44 were increased in Tregs cells and SMCs (Figure 6C). Decreasing signaling was observed as shown in Figure 6D. Subsequently, a pseudo-chronological analysis was performed with SMCs, which revealed different characteristics in SMCs. In addition, HNRNPA2B1 levels were observed to be different in different cell subsets, suggesting that HNRNPA2B1 could have a role in the functional regulation of SMCs (Figures 6E–H). The HNRNPA2B1 expression levels determined were consistent with the proliferation-related and collagen synthesis-related gene Col4a1 (Figure 6H).

### The HNRNPA2B1—lncRNA-miRNA-mRNA interaction could promote the PASMCs phenotypic switch

**FIGURE 7:**
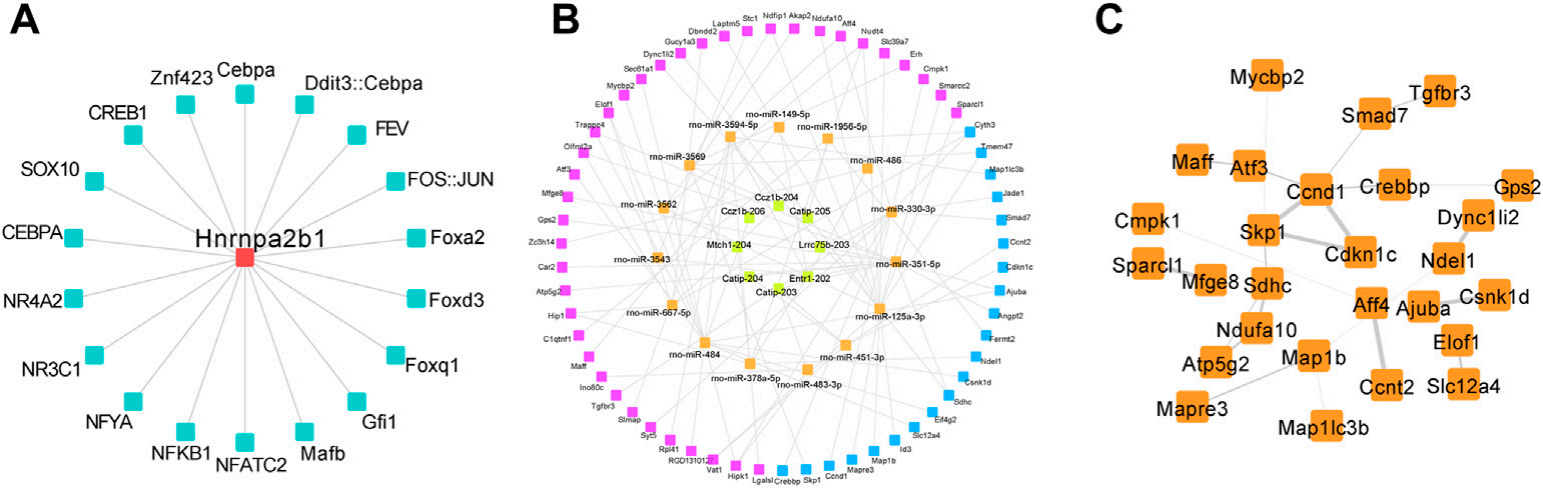
The HNRNPA2B1–lncRNA/miRNA/mRNA interaction could promote the PASMCs phenotypic switch. **(A)** The predicted TFs regulated HNRNPA2B1. **(B)** The lncRNA-miRNA-mRNA ceRNA network in the PASMCs. Yellow and orange nodes represent lncRNAs and miRNAs, respectively. Purple and blue nodes represent the mRNAs and the target genes of HNRNPA2B1, respectively. **(C)** The PPI network of the mRNAs in the ceRNA network.

The construction of a lncRNA—miRNA—mRNA ceRNA network based on HNRNPA2B1 in the present study allowed for identifying the regulatory relationship between HNRNPA2B1 and the ceRNA network, in addition to their roles in PAH development. The transcription factors potentially regulating HNRNPA2B1 were predicted, and the TOP 10 factors, included Mafb, NFATC2, NFYA, NFκB1, NR3C1, NR3A2, SOX10, CEBPA, and ZNF423. (Figure 7A). The lncRNA-miRNA-mRNA ceRNA network in the PASMCs indicated that rno-miR-330-3p/TGFbr3, rno-miR-125a-3p/slc39a1, and other combinations could contribute to SMCs proliferation and pulmonary remodeling (Figure 7B; Tables 1, 2). The PPI network of the mRNAs in the ceRNA network showed that TGFβ3/SMAD7 signaling might be a downstream mechanism of HNRNPA2B1 (Figure 7C).

**TABLE 1:** The top 8 lncRNAs predicted based on their involvement in the interaction between HNRNPA2B1 and miRNAs.

lncRNA	miRNA
Catip-203	rno-miR-351–5p, rno-miR-451–3p, rno-miR-483–3p
Catip-204	rno-miR-351–5p, rno-miR-451–3p
Catip-205	rno-miR-1956–5p, rno-miR-3569
Ccz1b-204	rno-miR-3594–5p, rno-miR-667–5p
Ccz1b-206	rno-miR-484
Entr1–202	rno-miR-125a-3p, rno-miR-149–5p, rno-miR-351–5p, rno-miR-3543, rno-miR-3562, rno-miR-3594–5p, rno-miR-378a-5p, rno-miR-486
Lrrc75b-203	rno-miR-330–3p
Mtch1–204	rno-miR-378a-5p

**TABLE 2:** The miRNAs with their respective target mRNAs in the ceRNA network.

miRNAs	mRNAs
rno-miR-330–3p	Aff4, Ccnd1, Cmpk1, Crebbp, Cyth3, Hipk1, Jade1, Smad7, Syt5, Tgfbr3
rno-miR-351–5p	Atp5 g2, Car2, Gps2, Mfge8, Olfml2a, Sdhc, Trappc4, Ajuba, Angpt2
rno-miR-125a-3p	Dync1 li2, Gucy1a3, Id3, Laptm5, Map1b, Mapre3, Ndfip1, Ndufa10, Nudt4, Sdhc, Slc39a7, Smarcc2, Sparcl1, Tmem47, Vat1
rno-miR-451–3p	Ccnt2, Cdkn1c, Vat1, Ccnt2, Cdkn1c, Vat1
rno-miR-483–3p	Hipk1, Ino80c
rno-miR-378a-5p	Hip1, Ndel1, Nudt4
rno-miR-484	Ajuba, Atf3, Csnk1d, Eif4 g2, Elof1, Hipk1, Mycbp2, Sec61a1, Slc12a4, Trappc4
rno-miR-667–5p	Cyth3, Dbndd2, Elof1, Hip1, Olfml2a
rno-miR-3543	Stc1
rno-miR-3562	Akap2, Nudt4
rno-miR-3569	Erh, Tmem47, Ndfip1, Skp1
rno-miR-3594–5p	Aff4, Lgalsl, Map1 lc3b, RGD1310127, Rpl41, Slmap
rno-miR-149–5p	Maff
rno-miR-1956–5p	C1qtnf1, Cyth3, Fermt2
rno-miR-486	Zc3 h14

### HNRNPA2B1 was significantly elevated in the PASMCs isolated from IPAH patients

**FIGURE 8:**
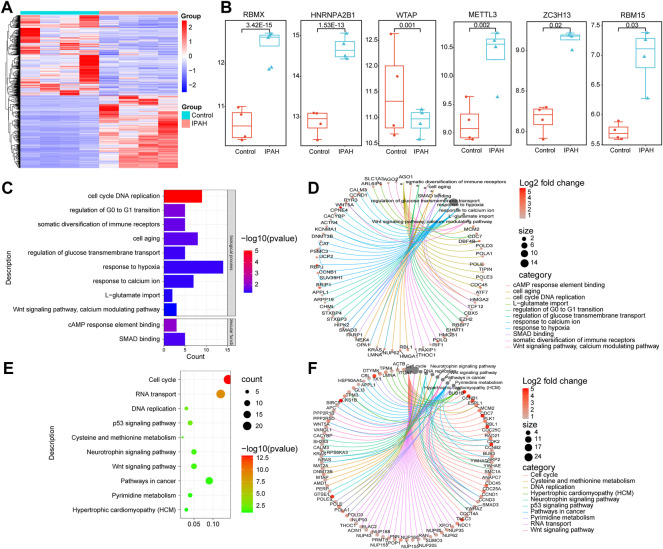
HNRNPA2B1 was significantly elevated in the pulmonary artery SMCs isolated from IPAH patients. **(A)** The heatmap of the differentially expressed genes in the datasets. **(B)** The differences in the expressions of m6A regulators. **(C)** The GO analysis of the target genes of m6A that overlapped with the upregulated DEGs. **(D)** The genes involved in these functions are presented. **(E)** The results of the KEGG analysis of the target genes of HNRNPA2B1 overlapped with the upregulated DEGs. **(F)** The genes involved in these functions are presented.

The validation of the expression of m6A regulators in the PASMCs isolated from IPAH patients was performed next. The heatmap of the DEGs in the PASMCs of IPAH and controls is depicted in Figure 8A. The validation analysis revealed that HNRNPA2B1, METTL3, ZC3H13, RBM15, and RBMX were increased, and WTAP was decreased in the PASMCs (Figure 8B). HNRNPA2B1 was significantly elevated in the PASMCs of IPAH patients, which was consistent with the scRNA analysis data (Figure 3A). The bioinformatics analysis of the overlapped genes between the upregulated DEGs and the target genes of HNRNPA2B1 indicated that HNRNPA2B1 could be involved in the regulation of the cell cycle of SMCs and also in cAMP, P53 signaling, and Wnt signaling pathways etc. (Figures 8C,E). The genes involved in the above functions are presented in Figures 8D,F.

### HNRNPA2B1 was highly expressed in the lung tissue of PAH

**FIGURE 9:**
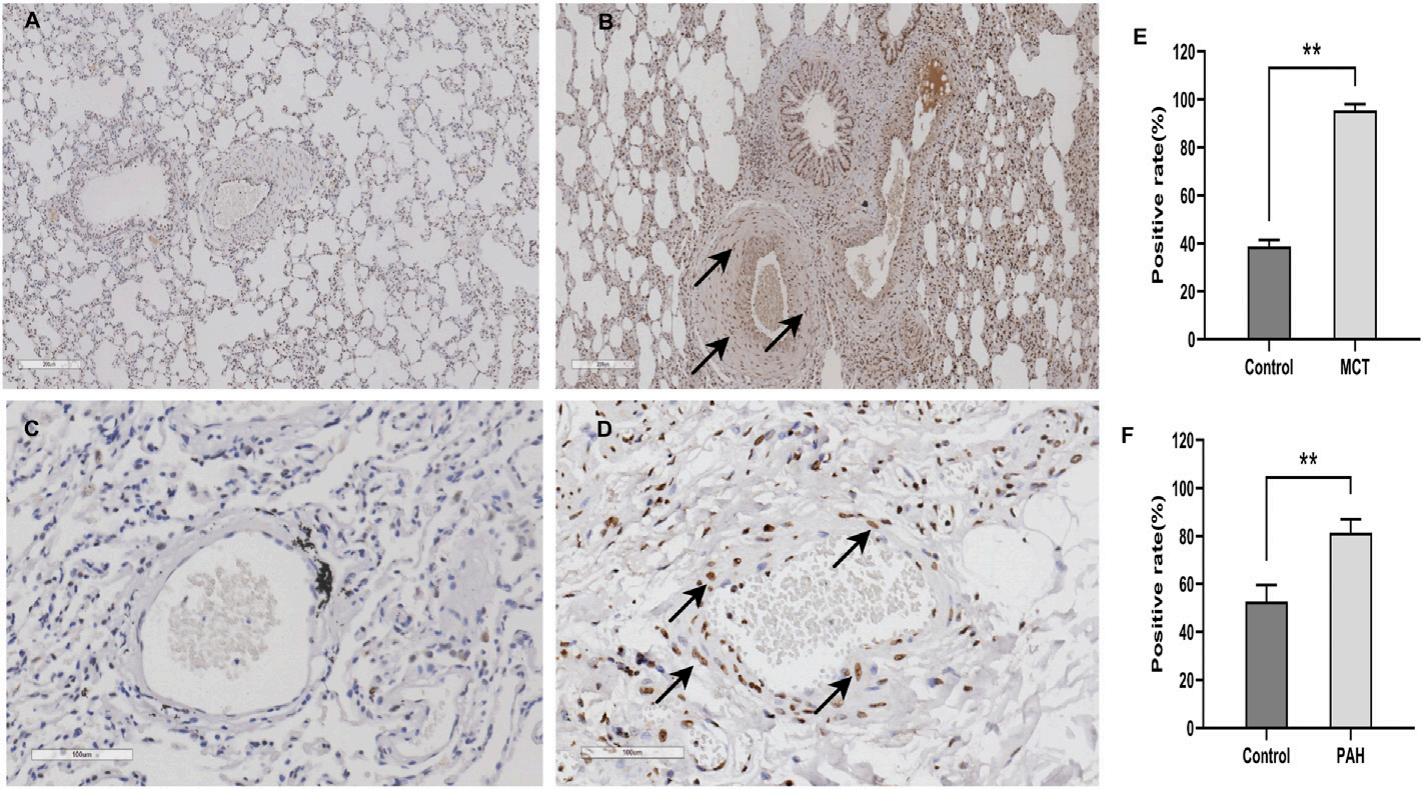
HNRNPA2B1 was highly expressed in the lung tissue of both MCT-PAH rat models and PAH patients. **(A,B)** HNRNPA2B1 was highly expressed in the MCT-PAH lung tissue **(B)** compared with controls**(A)**, as determined using IHC(×200). **(C,D)** HNRNPA2B1 was highly expressed in the human PAH lung tissue **(D)** compared with healthy donors**(C)**, as determined using IHC (×200). **(E,F)** Quantification data was showed in E and **(F)** (**p<0.01).

The results of the bioinformatics analysis suggest that HNRNPA2B1 was significantly elevated in pulmonary hypertension and plays an important functional role in SMCs. The significantly high expression of HNRNPA2B1 in the lung sample of MCT-PAH (Figure 9B) compared with controls (Figure 9A) was confirmed using immunohistochemistry. (*p* ‹ 0.01) In addition, HNRNPA2B1 levels in the lung tissue of patients with PAH were determined, and the results were consistent. The expression of HNRNPA2B1was increased in PAH patients (Figure 9D) compared with healthy donors (Figures 9C,E,F) (*p*<0.01).

## Discussion

Despite substantial improvements in the diagnosis and treatment of PAH over the past decade, patients with PAH continue to exhibit a poor prognosis (Thenappan et al., 2018). A greater understanding of the mechanisms underlying PAH is critical for the development of further effective therapeutics against this condition (Ricachinevsky and Amantéa, 2020). Interactions between multiple cell types eventually lead to the development of PAH. ECs dysfunction, SMCs phenotypic switching, fibroblast activation, and immune cell disturbances are the major cell types involved in the occurrence and development of PAH (Tuder, 2017; Hassoun, 2021). Studies from the past 2 years have reported that RNA epigenetic modification, particularly the m6A methylation modification, was involved in the occurrence and development of pulmonary hypertension (Zhou et al., 2021a). Our analytical results demonstrated the dysregulation of multiple m6A regulators in the lung of MCT-PAH rats (Figure 1H), which was partly consistent with the findings reported in previous studies (Zeng et al., 2021). However, knowledge regarding the contribution of m6A regulators in mediating PAH-associated vascular remodeling, including ECs dysfunction, pulmonary vascular fibrosis, and immune dysregulation, remains unknown so far. In order to comprehensively analyze the differences in the m6A regulators in specific cell types and the underlying mechanisms, scRNA data of lung tissue samples from MCT-PAH rat models (http://mergeomics.research.idre.ucla.edu/PVDSingleCell/) were analyzed in the present study.

**FIGURE 10:**
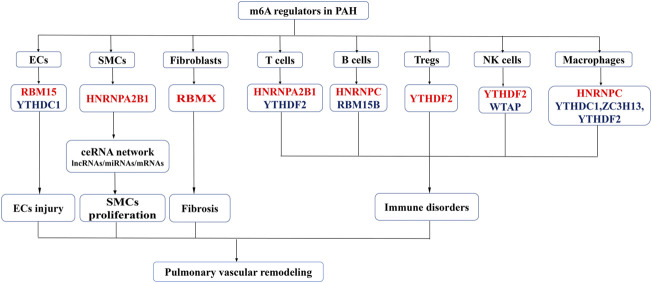
An overview of dysregulated m6A regulators and proposed mechanism of the study. Red represent the increased m6A regulators and the blue represent the decreased m6A regulators in PAH. M6A regulators promotes the phenotype switch in PASMCs proliferation, ECs injury, fibroblast activation and immune disorders leading to pulmonary vascular remodeling and PAH development.

The present study was, to the best of our knowledge, the first one to analyze the expression of m6A regulators in different cell subsets, including PASMCs, PAECs, fibroblasts, macrophages, NK cells, B cells, T cells, and Tregs (Figure 10). The dysfunctions of these cells collectively lead to pulmonary vascular remodeling in PAH (Hassoun, 2021; Poch and Mandel, 2021).

Endothelial cell dysfunction, inflammation, oxidative stress, and endothelial-mesenchymalization play critical roles in the vascular remodeling in pulmonary hypertension (Ou et al., 2020; Sharma et al., 2021). Recent studies have revealed that m6A regulators play a vital role in ECs injury and pathological proliferation in an m6A modification-dependent manner (Li et al., 2021). The results of the present study revealed changes in the m6Aregulators in the ECs of MCT-PAH. YTHDC1 levels were decreased while RBM15 levels were significantly upregulated. YTHDC1 is an m6A reader that regulates mRNA splicing and mRNA destabilization and also mediates the export of methylated mRNA from the nucleus to the cytoplasm (Xiao et al., 2016; Roundtree et al., 2017; Shima et al., 2017). Studies have demonstrated that YTHDC1 may promote tumorigenesis, cell proliferation, and cancer cell migration (Zhu et al., 2021b). Rui Xu et al. elucidated a novel role of YTHDC1 in brain injury, which involved promoting PTEN mRNA degradation and increasing Akt phosphorylation (Zhang et al., 2020b). However, the role of YTHDC1 in PAH remained unknown so far. The results of the bioinformatics analysis conducted in the present study revealed that YTHDC1 participated in ECs adhesion, proliferation, and migration. Therefore, it was inferred that studying the regulation of m6A regulators in ECs would assist in deciphering the mechanisms underlying ECs injury and pulmonary vascular remodeling.

Fibroblasts are activated through various pathways, and activated fibroblasts are characterized by excessive proliferation, apoptosis, promotion of inflammation, and metabolic reprogramming, which may cause the muscle fibroblasts to migrate to medium film or even the inner membrane, thereby promoting vascular wall thickening (Li et al., 2016). The fibroblasts also significantly alter the production and degradation of ECM proteins to participate in ECM remodeling. Fibroblasts release ROS, which participate in oxidative stress. Activated fibroblasts modulate their cancerous phenotype through metabolic reprogramming, thereby playing an important role in the pulmonary vascular proliferation in PAH (Zhang et al., 2017). In the present study, RBMX was observed to be significantly upregulated in MCT-PAH. Moreover, the bioinformatics analysis revealed that the m6A regulator target DEGs of fibroblasts were related to the glycolytic process, PDGF receptor binding, NFκB transcription factor activity, p53 and PI3K−Akt signaling pathways, cellular response to oxidative stress, cell−substrate adherens junction, and regulation of mitotic cell cycle (Figures 4B,D). Therefore, it was inferred that m6A modification participated in fibroblast activation and promotion of pulmonary vascular remodeling.

Perivascular inflammation reportedly plays a key role in pulmonary vasoconstriction and pulmonary vascular remodeling, which promotes the development and progression of pulmonary hypertension (Pullamsetti and Savai, 2017; Florentin et al., 2018). According to a study, m6A modification mediated immune responses *via* post-transcriptional regulators in cells (Liu C. et al., 2021). The results obtained in the present study revealed that m6A regulators were dysregulated in interstitial macrophages, NK cells, B cells, T cells, and Treg cells. Recent studies have reported abnormal Tregs activity associated with pulmonary vascular injury *via* the Treg/T17 axis (Tamosiuniene et al., 2018). The results revealed that the target m6A DEGs were related to the regulation of vascular SMCs proliferation, lactate metabolic process, response to oxidative stress, HIF−1 signaling pathway, and platelet activation in Tregs (Figures 5F,H). These results suggested that the targets of m6A regulators participated in the regulation of the activity of Tregs. According to previous studies, abnormal Treg function was strongly correlated with a predisposition to PAH in animal models and PAH patients (Tian et al., 2021). The present study was the first one to reveal that the m6A regulators in Tregs were abnormal, and the target genes of these m6A regulators were involved in the regulation of the important biological functions of Tregs. Future research should, therefore, focus on studying the m6A modification in Tregs to develop strategies for reversing pulmonary vascular remodeling and alleviating the progression of PAH.

Several recent studies have demonstrated that the inflammatory response of macrophages may promote the progression of PAH (Zhu et al., 2015; Viola et al., 2019; Mouton et al., 2020; Zhang et al., 2020a). Studies have also demonstrated that m6A modification regulates macrophage phenotypic activation (Yin et al., 2021) and macrophage reprogramming through the stabilization of TSC1 and PPARγ (Wang et al., 2021b). Therefore, targeting the m6A regulators in macrophages might inhibit inflammation infiltration in PAH through the inhibition of the inflammatory reaction of macrophages. These findings provide novel directions for the research on the role of m6A modifications in immune cell-mediated inflammation and pulmonary vascular remodeling in PAH. Previous study has shown that intercellular signaling mechanism in the PA adventitia controlled by fibroblast-mediated signaling shapes macrophage differentiation towards a distinct phenotype critically regulated through IL6, STAT3, HIF1, and C/EBPβ. (El Kasmi et al., 2014). Beyond that, metabolic reprogramming of macrophages causes macrophage polarization and releases inflammatory factors leading to fibroblast activation and pulmonary vascular fibrosis (Wang et al., 2021a). M6A regulators participated in phenotypic polarization of macrophages and fibroblast activation (Wang et al., 2021b; Zhang et al., 2021). The regulatory role and mechanism of m6A regulators in the interaction between macrophages and fibroblasts may have important implications for PAH diagnosis and treatment. According to the previous reports on SMCs, METTL3, and YTHDF1 play key roles in the regulation of SMCs proliferation (Qin et al., 2021a; Hu et al., 2021). The results of the present study revealed that HNRNPA2B1 levels were significantly increased in SMCs (Figure 3A), which was consistent with the results of total lung transcriptome analysis (Figure 1E). The HNRNPA2B1, RBMX, and RBM15 levels were also observed to be significantly elevated in the PASMCs of IPAH patients (Figure 8B). RBM15 knockdown decreased the expression of CASP3 in an m6A-dependent manner and inhibited human aortic SMCs apoptosis (Fu et al., 2022). RBM15 and RBMX facilitate the proliferation and invasiveness of tumors, such as hepatocellular carcinoma (Song et al., 2020; Cai et al., 2021). However, the mechanism of RBM15 and RBMX in PASMCs is unclear. The significantly high expression of HNRNPA2B1 in MCT-PAH was further confirmed by the results of immunohistochemistry analysis (Figure 9B). In addition, HNRNPA2B1 levels in the lung tissue of PAH patients were determined, and encouragingly, the results were consistent (Figure 9D). It is suggested that HNRNPA2B1 might be an important regulatory gene in the occurrence of PAH. Therefore, exploring whether the intervention of HNRNPA2B1 could alleviate pulmonary arterial remodeling is of great significance. According to previous studies, HNRNPA2B1, as an important mRNA processing regulator, plays an important role in lymphoma, tumors, inflammation, and other disease conditions (Guo et al., 2020; Jiang et al., 2021a; Tang et al., 2021a; Zhu et al., 2021a). The bioinformatics analysis conducted in the present study revealed that HNRNPA2B1 participated in the Wnt signaling pathway, muscle cell differentiation, and TGFβ signaling pathway (Figures 3B,D). Moreover, when the transcriptome analysis data of the PASMCs isolated from IPAH patients was analyzed, it was indicated that the target DEGs of HNRNPA2B1 were related to cAMP, p53, and Wnt signaling pathways, cell proliferation, and cell adhesion (Figures 8C,E). Therefore, it was inferred that HNRNPA2B1, as an m6A reader, interfered with mRNA splicing, transport, and maturation which mediate the phenotype transition of PASMCs.

The occurrence and development of PAH involve vascular remodeling, which is caused by multiple cells. Studies have revealed that during PAH development, CCR2 and CCR5 are required for collaboration between macrophages and PASMCs to initiate and amplify PASMCs migration and proliferation (Abid et al., 2019). However, knowledge regarding the other receptor–ligand pairs and the regulatory interactions between other cells in PAH remains scarce. The scRNA sequencing data provide technical evidence for revealing the mechanisms underlying this disease. Therefore, an analysis of cellular interactions was performed using this dataset. The screening of the receptor–ligand pairs regulated by HNRNPA2B1 enabled deciphering the important mechanisms underlying the role of HNRNPA2B1 in regulating cell interactions. The results of the analysis of SMCs interactions in PAH in the present study indicated that these cells interact with ECs *via* the NPR1/VEGFA, FLT1/VEGFA, and Col1a1/a1b1 complexes. In addition, Wnt5A/ANTXR1 and Wnt5A/FZD1 were related to the interactions between PASMCs and fibroblasts. Therefore, it was inferred that HNRNPA2B1 participated in pulmonary artery remodeling in an m6A-dependent manner through the regulation of receptor—ligand pairs and cell interactions. Studies have shown that CD44-dependent inflammation, and collagenolysis regulates extracellular matrix remodeling (Govindaraju et al., 2019). CD44 participated in the regulation of endothelial-to-mesenchymal transition (EndMT) in the neointimal layer of PAH affected pulmonary arterioles (Agrawal and Hemnes, 2019; Isobe et al., 2019). Our results reveal a novel mechanism by which CD44/COL4A1 signaling was involved in regulating ECs dysfunction. Moreover, COL1A1/CD44, COL4A1/CD44, FN1/CD44, LAMC1/CD44, and COL6A2/CD44 signaling related to the interactions between Interstitial macrophages and SMCs were increased in MCT-PAH compared with controls. Meanwhile, COL6A2/CD44, FN1/CD44 were increased in Tregs cells and SMCs. The results suggest other mechanisms that CD44 may play in pulmonary vascular remodeling, mediating immune regulation, regulating collagen synthesis, SMCs proliferation, and phenotypic transition. Syndecan-4 (SDC4) is a transmembrane (type I) heparan sulfate proteoglycan that binds to, and modulates the activity of many extracellular proteins related to cytoskeletal protein binding and fibronectin binding implicated in tumor development (Cai et al., 2020; Godmann et al., 2020). We first reported that COL1A1/SDC4, COL6A2/SDC4 signaling related to the interactions between Interstitial macrophages and SMCs were increased. SDC4 has not been studied in pulmonary hypertension and may promote vascular remodeling by mediating collagen synthesis. Also, PTPRC—MRC1 signaling related to the interactions between NK cells and Fibroblasts was increased and more studies need to explore the mechanism in the development of PAH. Pseudo-time trajectory analysis was performed on SMCs at all time points to reproduce the process of cell reprogramming, and then the differences in gene expression in different branch cells and the dynamic changes of genes in the same branch cells were compared to find the key factors affecting the reprogramming process. The HNRNPA2B1 expression levels determined were consistent with the proliferation-related and collagen synthesis-related gene COl4A1 (Figure 6H). Therefore, it could be that HNRNPA2B1 promotes the phenotype switch of PASMCs.

Previous studies have demonstrated that HNRNPA2B1 may aggravate tumor progression by activating Wnt-β/catenin signaling *via* m6A (Wang et al., 2018; Rong et al., 2022). In the present study, the transcription factors potentially regulating HNRNPA2B1 were predicted, and the TOP 10 factors, including Mafb, NFATC2, NFYA, NFκB1, NR3C1, NR3A2, SOX10, CEBPA, and ZNF423, are presented in Figure 7A. These findings would provide novel directions for the further investigation of HNRNPA2B1 in future studies. Most of the identified TFs have not been reported previously and would, therefore, require further validation.

Accumulating evidence suggests that lncRNAs contribute to PASMCs proliferation and pulmonary vascular remodeling through a variety of pathways (Yang et al., 2019; Qin et al., 2021b; Song et al., 2021). The ceRNA hypothesis proposes that lncRNAs, as ceRNAs, regulate gene expression in PAH by competing for shared miRNAs (Liu et al., 2021a; Liu et al., 2021b). The m6A regulators were reported to be involved in the modification of non-coding RNAs (Huang et al., 2020). Studies have demonstrated that the interaction of lncRNA with HNRNPA2B1 facilitates an m6A-dependent stabilization of mRNA, which promotes the progression of colorectal cancer (Liu et al., 2022). In the present study, the interactions of HNRNPA2B1 with lncRNAs were predicted (Figure 7B; Table 1), and subsequently, the top 10 lncRNAs were selected for further analysis. The interactions between lncRNAs and miRNAs or mRNAs were integrated to construct a lncRNA–miRNA—mRNA ceRNA network, which allowed for exploring the mechanism of HNRNPA2B1. It is reported that miR-125a-3p is involved in the regulation of cell proliferation and apoptosis, thereby mediating tumorigenesis and development (He et al., 2020). The results of the present study demonstrated that rno-miR-330-3p/TGFβr3, rno-miR-125a-3p/slc39a1, and other combinations could be contributing to SMC proliferation and pulmonary remodeling. The construction of a lncRNA—miRNA—mRNA ceRNA network based on HNRNPA2B1 in the present study allowed for identifying the regulatory relationship between HNRNPA2B1 and the ceRNA network, in addition to their roles in PAH development. Moreover, the bioinformatics analysis revealed that ceRNA-related mRNAs were involved in important biological processes, such as DNA replication, cell cycle regulation, and phenotypic switch (Figure 7B). Therefore, it was inferred that HNRNPA2B1 regulated the biological processes and pathways of PASMCs *via* the lncRNA—miRNA—mRNA ceRNA network. The present study contributes to a better understanding of the pathogenesis mediated by HNRNPA2B1 and the role of the lncRNA—miRNA-mRNA network in disease progression. HNRNPA2B1 could serve as a novel target in the treatment of PAH. Interventions targeting m6A regulators would enable ameliorating PAH and reducing the phenotypic switching of PASMCs and pathological vascular remodeling, thereby serving as a novel effective strategy for the diagnosis and treatment of PAH.

This is the first systematical analysis of the expression of N6-Methyladenosine RNA methylation regulators in PAH. This study has several limitations, first of all, the study and conclusion came from bioinformatics analysis, most of them were theoretically validated, but not verified by experimental conditions, leading to the accuracy weakened. Secondly, there is no available relevant m6a2 target data for rats, whilst the GEO dataset and single-cell sequencing dataset used are rats based. Nevertheless, a certain amount of data and results were obtained from this study, which would be used by the academic community to carry out deeper research on m6A-related studies in the field of PAH. Last but not least, this study provided novel scientific evidence to a better understanding of PAH pathogenesis. In summary, the present study is a pioneer in reporting changes in the m6A regulators in PAH based on scRNA data and the bioinformatics analysis of PAECs, PASMCs, NKs, B cells, T cells, and Tregs in PAH. m6A Regulators could be promising biomarkers for the diagnosis and treatment of PAH. In the future, further intervention on m6A regulators would be of great significance as a novel strategy against the occurrence and development of PAH.

## Data Availability

The original contributions presented in the study are included in the article/supplementary material, further inquiries can be directed to the corresponding authors.
